# Traumatic brain injury hospitalizations in Belgium: A brief overview of incidence, population characteristics, and outcomes

**DOI:** 10.3389/fpubh.2022.916133

**Published:** 2022-08-08

**Authors:** Helena Van Deynse, Wilfried Cools, Bart Depreitere, Ives Hubloue, Carl Ilunga Kazadi, Eva Kimpe, Karen Pien, Griet Van Belleghem, Koen Putman

**Affiliations:** ^1^Interuniversity Centre of Health Economics Research, Vrije Universiteit Brussel, Brussels, Belgium; ^2^Interfaculty Center Data Processing and Statistics, Vrije Universiteit Brussel, Brussels, Belgium; ^3^Department of Neurosurgery, Universitair Ziekenhuis Leuven, Katholieke Universiteit Leuven, Leuven, Belgium; ^4^Department of Emergency Medicine, Universitair Ziekenhuis Brussel, Vrije Universiteit Brussel, Brussels, Belgium; ^5^Department of Medical Registration, Universitair Ziekenhuis Brussel, Brussels, Belgium

**Keywords:** traumatic brain injury, incidence, epidemiology, outcome, administrative data, population-based studies

## Abstract

**Background:**

There is a need for complete and accurate epidemiological studies for traumatic brain injury (TBI). Secondary use of administrative data can provide country-specific population data across the full spectrum of disease.

**Aim:**

This study aims to provide a population-based overview of Belgian TBI hospital admissions as well as their health-related and employment outcomes.

**Methods:**

A combined administrative dataset with deterministic linkage at individual level was used to assess all TBI hospitalizations in Belgium during the year 2016. Discharge data were used for patient selection and description of injuries. Claims data represented the health services used by the patient and health-related follow-up beyond hospitalization. Finally, social security data gave insight in changes to employment situation.

**Results:**

A total of 17,086 patients with TBI were identified, with falls as the predominant cause of injury. Diffuse intracranial injury was the most common type of TBI and 53% had injuries to other body regions as well. In-hospital mortality was 6%. The median length of hospital stay was 2 days, with 20% being admitted to intensive care and 28% undergoing surgery. After hospitalization, 23% had inpatient rehabilitation. Among adults in the labor force pre-injury, 72% of patients with mild TBI and 59% with moderate-to-severe TBI returned to work within 1 year post-injury.

**Discussion:**

Administrative data are a valuable resource for population research. Some limitations need to be considered, however, which can in part be overcome by enrichment of administrative datasets with other data sources such as from trauma registries.

## Introduction

Traumatic brain injury (TBI) is defined as “an alteration in brain function, or other evidence of brain pathology, caused by an external force” (1). In 2017, many of the world's leading researchers in the field of Traumatic Brain Injury (TBI) collaborated in the Lancet Neurology Commission to highlight research priorities. As noted in their report, TBI outcomes in high income countries have not substantially improved over the last decades (2). Amongst others, the authors attribute this lack of progress to policy factors. Also, they indicate an associated lack of political and public awareness of the burden TBI causes for individuals and society as a whole. Therefore, the first of nine chapters on research priorities in the report was devoted to the epidemiological domain and its need for complete and accurate population studies. These studies can contribute to the knowledge that is required to inform healthcare policy, including prevention strategies and to increase awareness (2). Epidemiological data are particularly important in high income countries given the changing patterns over the past few decades (3, 4). More specifically, increased road safety as well as a vastly aging TBI population have led to a decline in mortality from transport-related TBI, and a shift toward more fall-related TBI deaths (2–4). Population data are important to identify high-risk populations and to enable appropriate preventive and therapeutic interventions for these populations (2).

Among the available epidemiological studies in high income countries, large variation in incidence and mortality can be observed (5). Moreover, as noted by Leibson et al. (6), population data on all ages, sexes, severities and injury mechanisms are scarce in current literature. In order to implement more targeted and effective programs for prevention and patient management, specific estimates on regional impact and trends are required across the full spectrum of disease (7). Country-specific and population-based data can be obtained through secondary use of administrative data. Administrative data are defined as information that is routinely collected for the operation of administrative systems, including registration, transaction and record keeping, usually in the context of the delivery of a service or public sector agencies (8, 9). For Belgium, this approach has been used by Peeters et al. (10), who described the changing epidemiological patterns of TBI using discharge data. However, the most recent data in this work date back to 2012. Apart from being somewhat outdated, this also implies that the ninth edition of the International Classification of Diseases (ICD-9-CM) was still in use rather than the more detailed tenth edition. Moreover, data linkage between different administrative sources can provide further opportunities to characterize the TBI population and outcome assessment.

The current study utilized a combined dataset of administrative sources. This study provides a profound descriptive overview of the Belgian hospital admissions with TBI, without aiming to compare or explain findings. The study has an additional focus on healthcare-related outcomes and employment outcomes.

## Methods

### Study dataset

This study included all new TBI hospitalizations in Belgium during the year 2016. The population-based administrative dataset (approval references: B.U.N.143201940065, IVC/KSZG/19/230, IVC/KSZG/20/410) utilized in this study covers all TBI patients hospitalized in Belgium in 2016, and consisted of three data sources. First, discharge data from all hospitals in Belgium were consulted for data on acute hospitalization, including the diagnostic information required to identify the study population. Second, claims data gave insight in the care received, and provided healthcare-related follow-up information beyond hospitalization. Finally, social security data were added to include data on return to work as an important outcome after TBI. An individual deterministic linkage (using the national registry number) was required to identify the population of interest in the latter two databases.

### Discharge data

Three classification systems were used to reorganize the data provided by ICD-10-CM into meaningful clusters.

First, the US Centers for Disease Control and Prevention (CDC) matrix for injury diagnoses was used to classify injury diagnoses by nature and body region and was thereby essential for patient selection. To be included in the study, patients were required to have a principal or secondary diagnosis of TBI, indicated by the ICD-10-CM codes S02.0, S02.1-, S02.8, S02.91, S04.02, S04.03-, S04.04-, S06-, S07.1, in line with this framework (11). Cases of shaken infant syndrome (T74.4) were reported but not included in the main analysis due to the very different nature of this condition and associated ICD-coding. Two additional requirements were installed to avoid selecting subsequent admissions resulting from a pre-existing TBI: (1) a TBI diagnosis with a seventh digit A, B, or C for an initial encounter, (2) a visit to an emergency department. Cases fulfilling neither of these criteria were reviewed individually.

A second CDC framework was used to classify external causes by mechanism and intention (11, 12). For transport accidents, we made a slight adjustment by only distinguishing between accidents with and without involvement of a motor vehicle, regardless of whether the patient was in the vehicle or not.

Finally, ICD-10-CM codes were used to discern mild from moderate-to-severe TBI. When available, a Glasgow Coma Scale score determined this distinction in line with common practice in TBI research (2). In the absence of this information, Head Abbreviated Injury Score (AIS) and Loss Of Consciousness (LOC) were utilized as other indicators of injury severity, which both had to imply minor severity for the case to be considered mild TBI. Head AIS was calculated using the ICD Programs for Injury Categorization, with a score of one corresponding to minor severity (13). For LOC, a duration of <30 min was deemed to be mild.

### Claims data

Belgium has a national health insurance with a wide coverage of health services. Reimbursement is received through the compulsory health insurance funds, covering virtually 100% of the population (14, 15). The data of the health insurance funds are pooled in a national claims dataset, containing detailed information about all reimbursable healthcare services for a time period up to 1 year post-TBI. Some personal information was included as well, such as mortality and entitlement to additional reimbursement. The latter mainly depends on household income and was used as a proxy for socioeconomic status in this study.

### Social security data

For each adult between 18 and 65 years old, the baseline and 1-year post-injury socioeconomic position was established by combining data about (self-)employment, retirement, child allowances, and benefits for unemployment or disability. This socioeconomic position served a double purpose. First, it was used to select the cases who were in the labor force pre-injury. The labor force was defined as those either employed or unemployed, as opposed to those who are jobless and not looking for a job, such as students, pensioners, and homemakers. Second, baseline and 1-year post-injury employment status were derived. Among salaried employees, full and partial work resumption were distinguished, with partial resumption defined as a relative decrease of 20 percent of the person's pre-injury full-time equivalent. For self-employed entrepreneurs, the absence of an employment contract implies a lack of data on full-time equivalents. Finally, a distinction was made between medical incapacity to work and not working for any other reason.

## Results

### Population

An overview of the 2016 population of TBI hospitalizations is presented in Table 1. A cumulative incidence of 136 cases per 100,000 was found. In children and older adults, the majority of TBIs were attributable to falls with 65 and 84%, respectively. For adults, falls caused half of the TBI admissions, with transport accidents responsible for an additional 35%. Assault accounted for 11% of the adult cases, whereas intentionally inflicted injury was rare in the other age groups. Skull fractures were infrequent in all age categories, while remarkable age differences in the occurrence of focal and diffuse intracerebral injuries can be noticed across the life span. Diffuse intracranial injury was reported in a large majority of children (88%), half of the adult population and a minority of older adults (36%). Conversely, the proportion of patients with focal intracranial injury was a mere 5% in children, 29% for adults and up to 51% in older adults. For the latter group, this can be attributed to increased frequencies of subdural hemorrhage (24%) and subarachnoid hemorrhage (10%). While most TBIs were isolated in children, injuries to other body regions were reported in most adult and older adult cases. Unsurprisingly, non-TBI injuries in the head region were most common (39%). Finally, our dataset contained nine cases of shaken infant syndrome, all of whom survived until the end of the one-year follow-up period of this study.

**Table 1:** Age-stratified overview of demographic and injury characteristics.

	**Children** **(0–18 yrs.)**	**Adults** **(19–65 yrs.)**	**Older adults** **(>65 yrs.)**	**Total**
**Demographics**				
Frequency	5,775	5,718	5,593	17,086
Incidence	227/100,000	83/100,000	289/100,000	136/100,000
Gender (male)	3,392 (59%)	3,551 (62%)	2,514 (45%)	9,457 (55%)
Low socio-economic status	1,196 (21%)	1,349 (24%)	2,093 (38%)	4,638 (28%)
Living alone	9 (<1%)	1,689 (30%)	2,833 (51%)	4,531 (27%)
**Cause of injury**				
**Mechanism**				
Fall	3,768 (65%)	2,272 (48%)	4,227 (84%)	10,267 (60%)
Transport (MV)	405 (7%)	1,177 (25%)	400 (8%)	1,982 (11%)
Transport (no MV)	260 (5%)	457 (10%)	223 (4%)	933 (6%)
Struck by/against	642 (11%)	669 (14%)	147 (3%)	1,352 (9%)
**Intention**				
Unintentional	5,272 (91%)	4,475 (88%)	5,064 (99%)	14,811 (87%)
Assault	96 (2%)	538 (11%)	43 (1%)	662 (5%)
Self-harm	7 (2%)	49 (1%)	11 (<1%)	67 (<1%)
**TBI**				
**Severity**				
Mild	4,115 (71%)	2,280 (41%)	1,328 (23%)	7,723 (45%)
Moderate-to-severe	1,660 (29%)	3,268 (59%)	4,435 (77%)	9,363 (55%)
**Loss of consciousness (LOC)**				
No LOC	4,540 (79%)	2,861 (50%)	3,500 (63%)	10,901 (64%)
LOC <30 min	758 (13%)	1,233 (22%)	729 (13%)	2,720 (16%)
LOC > 30 min	28 (<1%)	197 (3%)	240 (4%)	465 (3%)
**Skull fracture**	165 (3%)	531 (9%)	375 (7%)	1,071 (6%)
**Focal intracranial injury**	275 (5%)	1,659 (29%)	2,875 (51%)	4,809 (28%)
Contusion	47 (1%)	269 (5%)	384 (7%)	700 (4%)
Epidural hemorrhage	55 (1%)	175 (3%)	122 (2%)	352 (2%)
Subarachnoid hemorrhage	28 (<1%)	394 (7%)	557 (10%)	979 (6%)
Subdural hemorrhage	85 (1%)	530 (9%)	1,327 (24%)	1,942 (11%)
Intracerebral hemorrhage	16 (<1%)	220 (4%)	424 (8%)	660 (4%)
**Diffuse intracranial injury**	5,098 (88%)	3,170 (55%)	2,006 (36%)	10,274 (60%)
Concussion	5,026 (87%)	2,928 (51%)	1,745 (31%)	9,699 (57%)
Diffuse axonal injury	63 (1%)	242 (4%)	260 (5%)	565 (3%)
**Other injuries**				
**Injuries in other body regions**	1,770 (31%)	3,738 (65%)	3,481 (62%)	8,989 (53%)
Non-TBI head, face, neck	1,398 (24%)	2,640 (46%)	2,578 (46%)	6,616 (39%)
Torso	198 (3%)	981 (17%)	753 (13%)	1,932 (11%)
Spine and back	56 (1%)	548 (10%)	391 (7%)	995 (6%)
Upper extremities	348 (6%)	1,139 (20%)	1,010 (18%)	2,497 (15%)
Lower extremities	261 (5%)	631 (11%)	590 (11%)	1,482 (9%)

All percentages are calculated relative to the full population within the specific age group. It can be noted that percentages of (cause of) injury variables do not add up to 100%. This is due to omission of non-informative categories of other, unspecified, or missing data.

### Outcomes

Table 2 contains mortality and healthcare-related outcomes of the previously described population. An overall acute mortality of 6% was found, with a substantially higher proportion for older adults (15%). The length of acute hospital stay had a median of 2 days (Q1 = 1, Q3 = 8) and increased with age. Intensive care admissions were similar for adults (28%) and older adults (26%), but much lower for children (5%). Intensive care stays had a median duration of 2 days (Q1 = 1, Q3 = 6), and less variation with age. Despite being rare in children, in-patient rehabilitation became more common with advancing age, especially in older adults (45%). A similar increasing tendency in inpatient rehabilitation length of stay could not be found. Frequencies for (neuro)surgery and mechanical ventilation during acute hospitalization were similar among adults and older adults, but clearly lower in children. Across the lifespan, moderate-to-severe TBI is systematically associated with higher mortality, more intensive care and rehabilitation admissions, and higher (neuro)surgery and ventilation rates.

**Table 2:** Overview of outcomes and healthcare utilization stratified by age and TBI severity.

	**Children (0–18 yrs.)**			**Adults (19–65 yrs.)**			**Older adults (**>**65 yrs.)**			**Total**		
**Severity**	**Mild**	**Mod.-Sev**.	**Mild-Sev**.	**Mild**	**Mod.-Sev**.	**Mild-Sev**.	**Mild**	**Mod.-Sev**.	**Mild-Sev**.	**Mild**	**Mod.-Sev**.	**Mild-Sev**.
**Mortality**												
In-hospital mortality	0 (0%)	14 (<1%)	**14 (<1%)**	5 (<1%)	202 (6%)	**207 (4%)**	41 (3%)	770 (18%)	**811 (15%)**	46 (<1%)	986 (11%)	**1,032 (6%)**
One year mortality	0 (0%)	14 (<1%)	**14 (<1%)**	24 (1%)	264 (8%)	**288 (5%)**	155 (12%)	1,348 (31%)	**1,503 (27%)**	179 (2%)	1,626 (17%)	**1,805 (11%)**
**Length of stay**												
**Acute hospitalization**	**4,115 (71%)**	**1,660 (29%)**	**5,775 (100%)**	**2,280 (41%)**	**3,268 (59%)**	**5,718 (100%)**	**1,328 (23%)**	**4,435 (77%)**	**5,593 (100%)**	**7,723 (45%)**	**9,363 (55%)**	**17,086 (100%)**
Mean (SD)	1 (2)	3 (9)	**2 (5)**	3 (6)	11 (24)	**7 (18)**	9 (14)	15 (18)	**14 (18)**	3 (7)	11 (19)	**8 (16)**
Median (Q1–Q3)	1 (1–1)	1 (1–2)	**1 (1**–**1)**	1 (1–3)	4 (2–10)	**3 (1**–**7)**	4 (2–11)	10 (4–19)	**8 (3**–**17)**	1 (1–2)	5 (1-13)	**2 (1**–**8)**
**Intensive Care**	**38 (<1%)**	**222 (13%)**	**260 (5%)**	**222 (10%)**	**1,389 (41%)**	**1,611 (28%)**	**140 (11%)**	**1,337 (31%)**	**1,477 (26%)**	**400 (5%)**	**2,948 (31%)**	**3,348 (20%)**
Mean (SD)	3 (9)	4 (7)	**4 (8)**	4 (6)	7 (10)	**6 (10)**	5 (8)	6 (9)	**6 (9)**	4 (7)	6 (10)	**6 (9)**
Median (Q1–Q3)	1 (1–2)	2 (1–3)	**2 (1–3)**	2 (1–3)	3 (1–7)	**2 (1–6)**	2 (1–3)	3 (1–7)	**3 (2–6)**	2 (1–3)	3 (2–6)	**2 (1–6)**
**Inpatient rehabilitation**	**54 (1%)**	**49 (3%)**	**103 (2%)**	**258 (11%)**	**786 (23%)**	**1044 (18%)**	**493 (39%)**	**2004 (46%)**	**2497 (45%)**	**805 (10%)**	**2839 (30%)**	**3644 (23%)**
Mean (SD)	(^*^)	44 (42)	**40 (42)**	38 (41)	66 (61)	**14 (39)**	49 (48)	49 (49)	**9 (29)**	45 (46)	55 (54)	**54 (53)**
Median (Q1–Q3)	(^*^)	29 (15–45)	**23 (10–45)**	27 (14–47)	46 (23–92)	**42 (22–85)**	36 (18–59)	35 (17–60)	**35 (17–59)**	35 (15–56)	37 (19–70)	**37 (19–68)**
**Acute care**												
Surgery	263 (6%)	304 (18%)	**567 (10%)**	606 (26%)	1,541 (46%)	**2,147 (38%)**	397 (31%)	1,640 (38%)	**2,037 (36%)**	1,266 (16%)	3,485 (37%)	**4,751 (28%)**
Neurosurgery	1 (<1%)	53 (3%)	**54 (1%)**	15 (<1%)	431 (13%)	**446 (8%)**	20 (2%)	549 (13%)	**569 (10%)**	36 (<1%)	1,033 (11%)	**1,069 (6%)**
Mechanical ventilation	4 (<1%)	73 (4%)	**77 (1%)**	28 (1%)	474 (14%)	**502 (9%)**	25 (2%)	436 (10%)	**461 (8%)**	57 (<1%)	983 (11%)	**1,040 (6%)**

* Due to an exceptional financial arrangement with the most important rehabilitation facility for children, our administrative dataset did not contain sufficient information on the rehabilitation length of stay (LOS) for many children. By consequence, we only had rehabilitation LOS data about one child, for which descriptive statistics could not be calculated.

**Figure 1:**
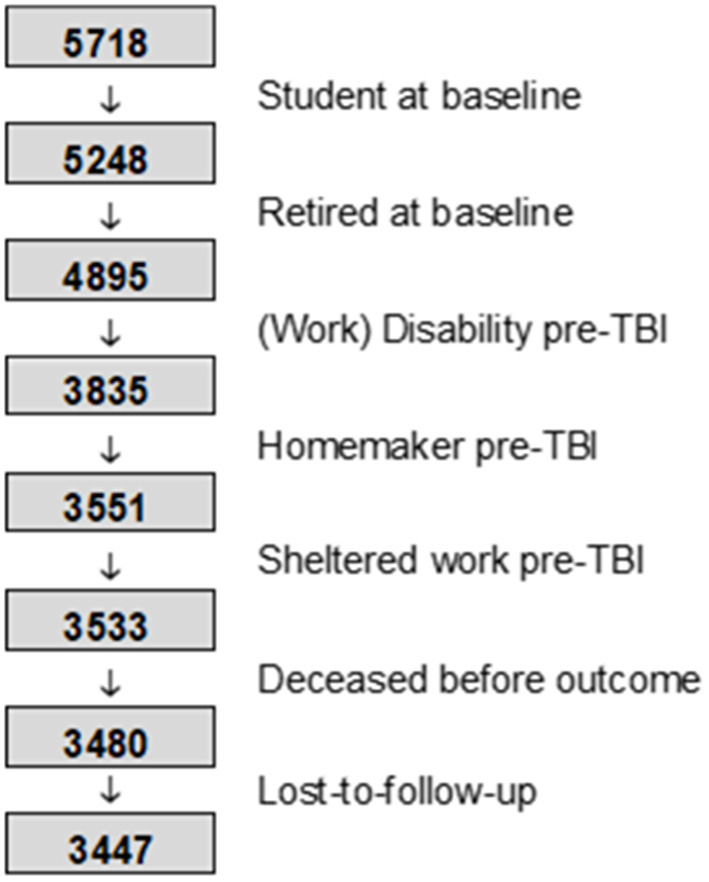
Patient selection for employment outcome assessment.

**Figure 2:**
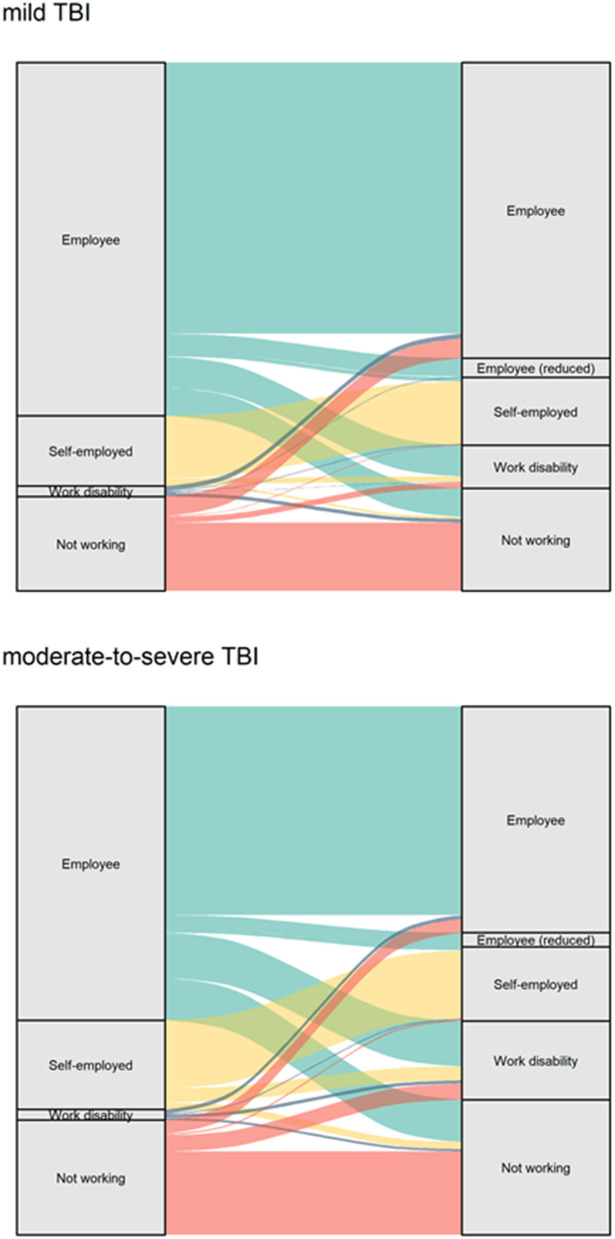
Alluvial plot displaying employment status pre-TBI and 1-year post-TBI.

A flow chart of the patient selection for the assessment of employment outcomes can be found in Figure 1. Figure 2 shows the transition between the pre-injury and 1 year post-injury employment status, separately for mild and moderate-to-severe TBI. In general, most patients return to their pre-injury situation. Among those employed for wage pre-injury, 77% of patients with mild TBI and 66% with moderate-to-severe TBI had a full return to work, with a similar small proportion for partial reinstatement in both groups (4 and 5%, respectively). More pre-injury employees were unemployed at 1 year post-TBI (work disability: 14%; jobless other reason: 13%) compared to the mild group (work disability: 9%; jobless other reason: 8%). Among those self-employed pre-TBI, 89% of mild TBI cases and 76% of moderate-to-severe cases resumed work. Overall, 20% of patients who were employed at the time of injury were no longer working at 1 year post-injury.

## Discussion

### General findings

This study found a total incidence of 136/100,000 TBI hospitalizations in Belgium in 2016. It must be noted that hospitalizations are not representative for the full population of TBI patients. In the EU, it has been estimated that hospitalizations only account for slightly more than half of the total number of cases. Moreover, hospitalized cases can be expected to be much more severe (2). For instance, this study found a slight majority with moderate-to-severe TBI, while the full TBI population has been estimated to consist of 70–90% mild TBIs (2).

As noted by the Lancet Neurology Commission, comparison of incidences and hospital admission rates across different studies and countries is very difficult, as the observed wide discrepancies in epidemiological findings are more likely to reflect methodological variation and hospital admission policies (2). The findings of this study did, however, follow the longitudinal tendencies established by Peeters et al. (10) regarding overall and age-specific admission rates, injury types, injury causes and mortality in Belgium. However, some international trends regarding epidemiology and demographics can be noted without interpreting absolute numbers. This study found a slight majority of male TBI patients, which is less pronounced than in earlier reports (3, 6, 7). The share of TBI patients with low SES (28%) was similar to previous findings, but exceeded that of the full 2016 Belgian population (19%) (3, 16). In line with current literature, this study found falls to be the predominant cause of injury across all age groups, followed by traffic accidents (2). The observed mortality is also within the range of available estimates (3, 17, 18).

### Age-specific findings

Despite children being the age group with the second largest incidence, they were overall the least severely injured. The large majority of admissions having mild TBI in children corresponds with the observed high frequency of concussions. Children had injuries to other body regions less often than other age groups. Additionally, regarding healthcare-related outcomes, children had shorter length of hospital stay and lower frequencies of intensive care, in-patient rehabilitation (neuro)surgery, and mechanical ventilation. The findings regarding concussions, other injuries and LOS are in line with previous studies (3, 7).

The most distinct characteristic of the adult population is injury cause, with notably higher shares of transport accidents and assault, as has been observed before (3, 7). An overall return to work rate for moderate-to-severe TBI of 59% was found. This is much higher than the 35% reported by Gormley et al. (19), which was aggregated from several studies with a sample that was not limited to those in the labor force pre-injury. Unsurprisingly, a higher return to work rate was found for mild TBI, with 72%.

In accordance with previous literature, the highest incidence and mortality were found for older adults (2, 20, 21). Mass lesions are known to be more common in older adults as they are often associated with fall-related TBI (22), which was observed to be the main cause of injury in this age group. Accordingly, focal TBIs were found to be more common with advancing age. In line with the typical injury pattern at old age, a remarkable increase in the occurrence of subdural hematomas can be noticed, though literature shows large variation in proportions (20, 23). Finally, the lack of informal care experienced by the large share of older adults living by themselves (51%) may in part explain the observed prolonged hospital stays and frequent admissions to in-patient rehabilitation as an increased reliance on formal care (24).

### Strengths and limitations

As administrative data are by definition gathered for operational purposes, they come with unique strengths and limitations, which have been discussed in earlier work (25–27). These limitations of administrative data imply that incidence figures derived from them should be interpreted with caution. Aside from discharge data only representing hospitalized patients, several studies comparing ICD-registrations for TBI diagnoses with other data sources (such as medical records) found that incidence of TBI-related admissions tends to be underestimated by discharge data, especially regarding mild TBI (28–32). The varying sensitivity of specific diagnostic ICD-codes reported in literature can explain some of our findings, such as the low incidence of contusions (32). The fact that discharge data are only available several years later, is another limitation. In this study, this time lag further increased due to COVID-related delays in data delivery and extensive data pre-processing. Finally, despite our efforts to exclusively consider acute cases of TBI in this work, it cannot be ascertained that some non-acute cases remained in our dataset by exception.

Nonetheless, administrative data are a valuable source of population-based data across the full spectrum of disease, which are scarce in current literature. They can provide a significant contribution to at least four out of the nine priority domains for TBI research identified by the Lancet Neurology Commission (2). Potential limitations of administrative data must be weighed against the opportunity cost of assembling the desired dataset, which involves time and resource intensive data collections (33). Therefore, the use of administrative data should ideally be supplemented with other methods (32). More relevant variables can for instance be found in a trauma registry. These also document emergency department visits without subsequent hospitalization, thereby providing more insight in the mild TBI population. Wynn et al. found trauma registry recordings to be more accurate than administrative data, which they attributed to coders' focus on trauma cases and close contact with physicians (31). Thus, linkage between administrative data and trauma registries provides additional opportunities for research to valorize the assets of administrative data while overcoming many of its downsides. Even though trauma registries are available in many countries with developed trauma systems, this is not the case for some countries, such as Belgium (34). In such a case, sensitivity analyses can provide more insight in overall and diagnosis-specific accuracy.

## Conclusion

This study provides a descriptive population overview of Belgian TBI hospitalizations during the year 2016. This may be used to prioritize targeted interventions for high-risk groups in terms of potential magnitude of impact. As an example, prevention of traffic injuries is most relevant in the adult population, while fall-prevention is particularly useful in the older adult population. This study also highlights areas for future research. For instance, the observation that 20% of the previously employed patients with TBI do not return to work, shows the need for more research on the determining factors of reinstatement after TBI. The results as presented can be used to support healthcare policies and initiatives to address the societal burden of TBI.

## Data availability statement

The datasets presented in this article are not readily available because the limitations imposed by the Belgian Data Protection Authority do not allow to share the study dataset because of privacy protection. Requests to access the datasets should be directed to contact@apd-gba.be.

## Ethics statement

This study protocol was reviewed and approved by the Ethical Committee of UZ Brussel (approval number B.U.N.143201940065) and the Belgian Data Protection Authority (approval numbers IVC/KSZG/19/230, IVC/KSZG/20/410).

## Author contributions

HV: conception, data preparation, data analysis, data visualization, initial draft of manuscript. WC: data visualization, review, and editing of manuscript. BD, IH, and KPi: medical supervision, review, and editing of manuscript. CI, EK, and GV: data preparation, review, and editing of manuscript. KPu: conception, review, and editing of manuscript. All authors contributed to the article and approved the submitted version.

## Funding

This study was funded by the Research Foundation Flanders (Grant number: 1S52420N) and the King Baudouin Foundation/Fund Benevermedex (Grant number: 2019-J5162090–214585). This study was published with support of the Belgian University Foundation (Grant number: WA-0413).

## Conflict of interest

The authors declare that the research was conducted in the absence of any commercial or financial relationships that could be construed as a potential conflict of interest.

## Publisher's note

All claims expressed in this article are solely those of the authors and do not necessarily represent those of their affiliated organizations, or those of the publisher, the editors and the reviewers. Any product that may be evaluated in this article, or claim that may be made by its manufacturer, is not guaranteed or endorsed by the publisher.
